# Patient adherence to and tolerability of self-administered interferon β-1a using an electronic autoinjection device: a multicentre, open-label, phase IV study

**DOI:** 10.1186/1471-2377-12-7

**Published:** 2012-03-05

**Authors:** Alessandra Lugaresi, Ciro Florio, Vincenzo Brescia-Morra, Salvatore Cottone, Paolo Bellantonio, Marinella Clerico, Diego Centonze, Antonio Uccelli, Maria di Ioia, Giovanna De Luca, Andrea Marcellusi, Andrea Paolillo

**Affiliations:** 1 Department of Neuroscience and Imaging, University "G. d'Annunzio", Via dei Vestini 31, 66100 Chieti, Italy; 2 Multiple Sclerosis Regional Center, Azienda Ospedaliera "Antonio Cardarelli", Via Antonio Cardarelli 9, 80131 Naples, Italy; 3 Department of Neurological Science, University of Naples Federico II, Via Giovanni Paladino 39, 80138 Naples, Italy; 4 Department of Neurology, Azienda Ospedaliera Ospedali Riuniti Villa Sofia-Cervello, Via Ninni Cassarà, 90146 Palermo, Italy; 5 Department of Neurology, Istituto Mediterraneo di Neuroscienze, NEUROMED, Via Atinense 18, 86077 Pozzilli, IS, Italy; 6 Division of Neurology, Department of Clinical and Biological Science, University of Turin, Via Giuseppe Verdi 8, 10124 Turin, Italy; 7 Department of Neuroscience, University of Rome "Tor Vergata", Via Montpellier 1, 00133 Rome, Italy; 8 Department of Neuroscience, Ophthalmology and Genetics, University of Genoa, Via De Toni 5, 16132 Genoa, Italy; 9 CEIS Sanità (CHEM-Centre for Health Economics and Management), Faculty of Economics, University of Tor Vergata, Via Columbia 2, 00133 Rome, Italy; 10 Merck Serono S.p.A., Via Casilina 125, 00176 Rome, Italy; 11 Department of Neuroscience and Imaging, University G. d'Annunzio, c/o Centro Sclerosi Multipla, Ospedale Clinicizzato "SS Annunziata", Via dei Vestini 31, 66100 Chieti, Italy

**Keywords:** Relapsing-remitting multiple sclerosis, IFN beta, Medication adherence, Drug delivery systems, Self administration

## Abstract

**Background:**

Achieving good adherence to self-injected treatments for multiple sclerosis can be difficult. Injection devices may help to overcome some of the injection-related barriers to adherence that can be experienced by patients. We sought to assess short-term adherence to, and tolerability of, interferon (IFN) β-1a administered via electronic autoinjection device in patients with relapsing-remitting multiple sclerosis (RRMS).

**Methods:**

BRIDGE (RebiSmart to self-inject Rebif serum-free formulation in a multidose cartridge) was a 12-week, multicentre, open-label, single-arm, observational, Phase IV study in which patients self-administered IFN β-1a (titrated to 44 μg), subcutaneously (sc), three times weekly, via electronic autoinjection device. Patients were assessed at baseline and 4-weekly intervals to Week 12 or early termination (ET) for: physical examinations; diary card completion (baseline, Weeks 4, 8 only); neurological examinations (baseline, Week 12/ET only); MS Treatment Concern Questionnaire (MSTCQ; Weeks 4, 8, 12 only); Convenience Questionnaire (Week 12 only); Hospital Anxiety and Depression Scale (HADS); and Paced Auditory Serial Addition Task (PASAT; baseline only). Adherence was defined as administration of ≥ 80% of scheduled injections, recorded by the autoinjection device.

**Results:**

Overall, 88.2% (105/119; intent-to-treat population) of patients were adherent; 67.2% (80/119) administered all scheduled injections. Medical reasons accounted for 35.6% (31/87) of missed injections, forgetfulness for 20.6% (18/87). Adherence did not correlate with baseline Expanded Disability Status Scale (*P *= 0.821) or PASAT (*P *= 0.952) scores, or pre-study therapy (*P *= 0.303). No significant changes (baseline-Week 12) in mean HADS depression (*P *= 0.482) or anxiety (*P *= 0.156) scores were observed. 'Overall convenience' was the most important reported benefit of the autoinjection device. Device features associated with handling and ease of use were highly rated. Mean MSTCQ scores for 'flu-like' symptoms (*P *= 0.022) and global side effects (*P *= 0.002) significantly improved from Week 4-12. Mean MSTCQ scores for pain at injection site and injection pain increased from Week 4-12 (*P *< 0.001). Adverse events were mild/moderate. No new safety signals were identified.

**Conclusion:**

Convenience and ease of use of the autoinjection device may improve adherence and, therefore, outcomes, in patients with RRMS receiving sc IFN β-1a.

**Trial registration:**

EU Clinical Trials Register (EU-CTR; http://www.clinicaltrialsregister.eu): 2009-013333-24

## Background

Multiple sclerosis (MS) is a chronic, inflammatory, degenerative, autoimmune disorder of the central nervous system with onset usually in young adulthood [1]. Most patients (70-80%) present with the relapsing-remitting form (RRMS), in which disability is accrued through incomplete recovery from intermittent disease relapses [1,2]. Approximately 50% of patients with RRMS develop disease progression within 10 years of disease onset [1,2].

There is currently no cure for MS. RRMS is treated primarily with established disease-modifying drugs (DMDs) aimed at reducing relapses. Current first-line DMDs include interferon (IFN) β and glatiramer acetate (GA), which are self-administered by subcutaneous (sc) or intramuscular (im) injection. Treatment of MS is a long-term commitment and outcomes may be influenced by the level of adherence to DMDs. Premature discontinuation and poor adherence have been associated with increased risk of relapse and disability progression [3-7], and may be due to adverse events (AEs) associated with the drug and route of administration (injection-site reactions [ISRs]) [8], 'flu-like' symptoms (FLS), perceived lack of efficacy, treatment fatigue, pain at injection site, injection anxiety [3,9-11] or, simply, forgetfulness. In some patients, forgetfulness and treatment fatigue may be exacerbated by cognitive deficits, depression and anxiety associated with MS [3,12]. Patients with MS may also experience impaired dexterity, reducing the ability to self-inject [2].

Strategies are needed to assist patients and healthcare providers to overcome and monitor adherence issues. Autoinjection devices can improve the injection experience and increase patient satisfaction [13-16], which could lead to improved adherence and treatment outcomes [17]. An electronic, handheld, multidose, autoinjection device that incorporates a dosing history log has been developed to improve the injection experience, patient satisfaction and treatment adherence among patients self-administering sc IFN β-1a. In a multicentre, international user trial, this device was considered 'very suitable' or 'suitable' for self-injection by 71.6% of patients and 92.2% reported some degree of suitability [14]. The objective of the study reported here was to assess short-term treatment adherence and tolerability in patients with RRMS who switched to sc IFN β-1a administered using the autoinjection device, either from other injectable DMD formulations or from sc IFN β-1a administered using a different injection system.

## Methods

BRIDGE (RebiSmart to self-inject Rebif serum-free formulation in a multidose cartridge [EudraCT number: 2009-013333-24]) was a 12-week, multicentre, open-label, single-arm, observational, Phase IV Italian study. Patients were recruited from 17 treatment centres across Italy between September 2009 and May 2010. The study was performed in accordance with the Note for Guidance on Good Clinical Practice (International Conference on Harmonisation of Technical Requirements for Registration of Pharmaceuticals for Human Use, Topic E6, 1996 and EU GCP Directive 2001/20/EC) and the guiding principles of the Declaration of Helsinki, and was approved by the Independent Ethics Committee/Institutional Review Board at each participating institution.

### Patients

Patients aged 18-65 years were eligible to participate if they had RRMS (according to revised 2005 McDonald criteria [18]); were eligible for sc IFN β-1a treatment (Rebif; Merck Serono S.A. — Geneva, Switzerland, a branch of Merck Serono S.A., Coinsins, Switzerland, an affiliate of Merck KGaA, Darmstadt, Germany), 44 μg three times weekly [tiw]); were switching from another injectable DMD or sc IFN β-1a using a different injection system; were able to self-inject using the autoinjection device (RebiSmart; Merck Serono S.A. — Geneva, Switzerland); were willing and able to adhere to the protocol for the duration of the study; and had provided written informed consent. Exclusion criteria included any other disease that may more adequately explain patient's signs and symptoms; use of immunosuppressive agents within 3 months of baseline; relapse within 30 days of baseline; pregnancy or breast-feeding, or refusal by patients who were not post-menopausal or surgically sterile to use a highly effective method of contraception throughout the study; elevated liver enzyme levels; inadequate bone marrow reserve; moderate-to-severe renal impairment; any visual impairment preventing self-injection with the autoinjection device; hypersensitivity to IFN or to any excipients; contraindications to ibuprofen; participation in any other investigational trial within 30 days of baseline; and any other significant disease that, in the opinion of the recruiting physician, would contraindicate participation in the trial.

### Study design

During the screening period, demographic data, medical history (including history of MS), blood and urine samples, and details of concomitant medications/procedures, medical conditions (safety assessment) and contraceptive methods were collected. Patients underwent physical and neurological examinations, a serum pregnancy test (where appropriate), and review of inclusion/exclusion criteria. Patients could withdraw from the study at any time. Upon withdrawal, all assessments required at the early termination (ET) visit were completed at the earliest time possible.

Patients underwent five study assessments over a 12-week period (Additional file 1: Figure S1): baseline (Visit 1/Study Day 1, conducted within 14 days of screening completion), Visit 2 (Week 2; telephone call), Visit 3 (Week 4), Visit 4 (Week 8) and Visit 5 (Week 12/ET). Treatment began at Visit 1 following completion of all baseline evaluations and patient training in the use of the autoinjection device.

### Interventions

Patients were provided with pre-filled cartridges, each containing three doses (44 μg/0.5 mL) of the serum-free (without foetal bovine or human serum albumin excipients) formulation of IFN β-1a, for self-injection sc tiw, using the electronic autoinjection device. IFN β-1a dose was titrated up over a 4-week period (8.8 μg/0.1 mL for the first 2 weeks, 22 μg/0.25 mL during Weeks 3 and 4, followed by the full dose of 44 μg/0.5 mL from Week 5 to 12). The study drug was to be administered at approximately the same time each day, on the same 3 days of the week, with a minimum of 48 h between injections. Patients were advised to rotate injection sites and to avoid injecting into inflamed areas. Dose reduction was not permitted and resulted in withdrawal of the patient from the study. Ibuprofen (400 mg prior to each injection and additional ibuprofen as necessary to maximum of 1200 mg/day) for the treatment of FLS was mandatory for the first 4 weeks of the study, after which ibuprofen was to be taken as required, according to the investigator's medical practice dosing schedule.

### Assessments

The assessment schedule is summarized in Additional file 1: Figure S1. Concomitant medications and AEs were recorded at all study visits. Patients underwent a physical examination at baseline and Weeks 4, 8 and 12/ET. A neurological examination, including Expanded Disability Status Scale (EDSS) score, was performed at baseline and Week 12/ET. Cognitive function was assessed at baseline using the Paced Auditory Serial Addition Task (PASAT), a serial addition task used to assess information processing speed, working memory and sustained attention on a scale of 0-60 (higher score indicating better cognitive function). Clinically significant depression and anxiety were assessed using the Hospital Anxiety and Depression Scale (HADS) at baseline and Weeks 4, 8 and 12/ET. Patients completed the MS Treatment Concern Questionnaire (MSTCQ) at Weeks 4, 8 and 12 to report FLS, ISRs and global side effects (GSEs), as well as the most important benefit of the autoinjection device from a list of five options, pain over the past 4 weeks (visual analogue scale [VAS]) on a scale of 0 (no pain) to 100 (worst possible pain), and pain upon injection during past 4 weeks on a scale of 1 (no pain) to 5 (horrible, excruciating pain). Reasons for missed injections were recorded using patient diary cards at baseline, Week 4 and Week 8. Patients also completed a Convenience Questionnaire (scale of 0-5, where lower scores indicate greater convenience) at Week 12 to provide an overall evaluation of the autoinjection device.

### Study endpoints

The primary endpoint was the proportion of patients at Week 12 who were adherent to treatment, defined as having administered ≥ 80% of scheduled injections over the course of the study, as logged by the autoinjection device. Patients who administered < 80% of scheduled injections were considered non-adherent. Adherence was calculated as 100 × (number of injections the patient administered)/(expected number of injections) over the 12-week study period. The 80% cut-off was chosen in accordance with previous studies on adherence using medication possession ratio (MPR) [19-22]. Secondary endpoints included changes in MSTCQ scores for side effects, device benefits and injection pain at Weeks 4, 8 and 12; change from baseline in HADS scores at Weeks 4, 8 and 12; overall evaluation of device convenience at Week 12; correlation of adherence at Week 12 with baseline PASAT score; frequency and reasons for premature discontinuation; and reasons for missed injections. Safety endpoints included treatment-emergent adverse events (TEAEs), including serious adverse events (SAEs), abnormal laboratory parameters and physical examination findings, and any concomitant medications and procedures.

### Analysis populations

The intent-to-treat (ITT) population was defined as all enrolled patients who had been assigned the autoinjection device. The safety population included all patients in the ITT population who had received at least one dose of the study drug.

### Statistical analyses

No hypothesis testing was conducted. A total of 120 patients were required to provide a 95% confidence interval (CI) equal to the sample proportion with 7% precision, based on the hypothesis that 80% of patients using the autoinjection device for 12 weeks would administer 80% of scheduled injections.

For the primary endpoint, the proportion of patients adherent to treatment in the ITT population and corresponding 95% CI was calculated. For the secondary endpoints, changes in adherence and VAS score over time were analysed using repeated-measures analysis of variance (ANOVA). Changes in ordinal scores over time (MSTCQ side-effect subscales, HADS, ratings) were analysed using the Friedman non-parametric test. Descriptive statistics were reported for device benefits, results of the Convenience Questionnaire, reasons for missed injections and reasons for withdrawal from the study. A Wilcoxon test was used to analyse change from baseline in EDSS scores. The predictive value of PASAT to assess non-adherence was analysed using the Student *t*-test and linear regression, or Spearman's ρ test as appropriate.

Logistic regression analyses using a forward stepwise method were performed to identify predictors of adherence, incorporating age, sex, disease duration, previous treatment, relapse frequency, and EDSS, PASAT and HADS scores. A *post hoc *analysis using Pearson's correlation was performed to investigate the relationship between HADS scores and FLS, ISRs and GSEs. Further *post hoc *analyses were performed to examine adherence in patients who had completed the study, creating the completers' population. Adherence in the completers' population was calculated as 100 × (number of administered injections)/(expected number of planned injections before withdrawal). Application of this analysis to the ITT population was also performed to assess adherence, with adjustments for missed injections due to withdrawals. Statistical significance was set at a level of 5%. Statistical analyses were performed using SPSS for Windows, version 18 (Chicago, IL, USA, 2002).

## Results

### Patients

**Figure 1:**
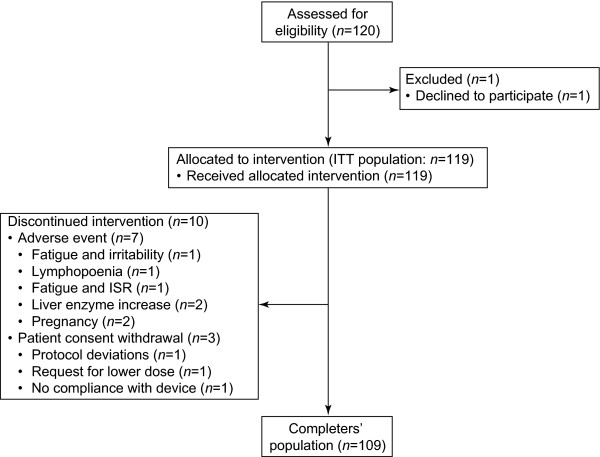
**Patient disposition**. ISR, injection-site reaction; ITT, intent-to-treat.

A total of 120 patients were recruited: 119 were included in the ITT population, 10 prematurely discontinued treatment and 109 completed the study (completers' population) (Figure 1). One patient withdrew from the study prior to administration of the first dose of study drug (Visit 1) and was excluded from the ITT population.

Patients were predominantly women (75.6% [90/119]), with a mean (standard deviation [SD]) age of 37.9 (9.68) years, a mean (SD) MS duration of 5.8 (5.31) years and a mean (SD) EDSS score of 2.1 (1.2; median: 2, range 0-6) (Additional file 1: Table S1). Prior to the study, most patients were receiving im IFN β-1a (63.0% [75/119]); only 2.5% (3/119) switched from sc IFN β-1a administered using a different injection system (Table 1).

**Table 1:** Baseline characteristics according to pre-study DMD treatment (intent-to-treat population; n = 119)

	Number (%) of patients	Age, years	Duration of MS, years	EDSS score*	PASAT score^†^	HADS Anxiety score^‡^	HADS Depression score^‡^
		
Pre-study DMD		Median (range); 95% confidence interval					
im IFN β-1a	75 (63.0)	39	5	2^¶^	42.5	7	5
		(19-58)	(0-22)	(0-5.5)	(5-60)	(0-20)	(0-20)
		35.6, 39.9	4.9, 7.3	1.9, 2.4	40.0, 45.3	6.9, 9.2	4.9, 7.0
IFN β-1b	26 (21.8)	37	3.5	1.5	48	7	3.5
		(20-52)	(0-22)	(0-6)	(24-59)	(0-13)	(0-15)
		34.1, 42.4	3.1, 8.6	1.3, 2.4	42.6, 50.8	5.0, 7.8	3.5, 7.1
Glatiramer acetate	15 (12.6)	41	4	1.5	35	7	3
		(18-55)	(1-8)	(0-4.5)	(18-52)	(0-14)	(0-12)
		31.7, 44.5	2.7, 5.3	1.3, 2.9	30.5, 41.9	3.8, 9.0	2.3, 7.0
sc IFN β-1a	3 (2.5)	35	6	3	46	4	6
		(22-43)	(6-11)	(2-4)	(25-51)	(4-10)	(2-6)
		7.0, 59.6	0.5, 14.8	0.5, 5.5	6.4, 74.9	-2.6, 14.6	-1.1, 10.4

*Range: 0 (no disability) to 10 (severe disability resulting in death)

^†^Range: 0 to 60 (higher scores indicate better cognitive function)

^‡^Range: 0 to 21 (higher scores indicate more severe symptoms)

^¶^n = 72

*DMD *disease-modifying drug, *EDSS *Expanded Disability Status Scale, *HADS *Hospital Anxiety and Depression Scale, *IFN *interferon, *im *intramuscular, *MS *multiple sclerosis, *PASAT *Paced Auditory Serial Addition Task, *sc *subcutaneous

### Treatment adherence

**Figure 2:**
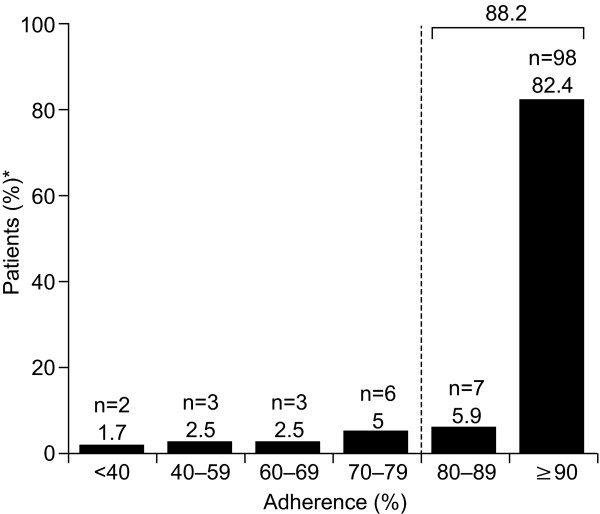
**Patient adherence at Week 12 in the intent-to-treat population (n = 119)**. *Percentages may not add up to 100 due to rounding.

Overall, 88.2% (105/119) of patients administered ≥ 80% of scheduled injections over the 12-week study period (ITT population; Figure 2). Treatment adherence was similarly high in women (87.8% [79/90]) and men (89.7% [26/29]). No significant correlation between adherence and baseline EDSS score (*P *= 0.821) or pre-study DMD (*P *= 0.303) was observed. Overall, the proportions of patients adherent to treatment stratified by pre-study DMD were 84.0% (63/75; im IFN β-1a), 93.3% (14/15; GA), 96.2% (25/26; sc IFN β-1b) and 100% (3/3; sc IFN β-1a). The proportion of patients who were adherent to treatment (i.e. administered ≥ 80% of scheduled injections) decreased significantly over time (*P *< 0.001; repeated measures ANOVA) from 99.2% (118/119) at Week 4 to 95.6% (114/119) at Week 8 and 81.5% (97/119) at Week 12. Sixty-seven percent of patients (80/119) administered all (100%) scheduled injections over the 12-week study; however, this proportion decreased significantly over time (*P *< 0.01) from 99.2% (118/119) at Week 4 to 95.0% (113/119) at Week 8 and to 67.2% (80/119) at Week 12.

In the completers' population, 96.3% (105/109) of patients were adherent to treatment, although a significant decrease in adherence was observed over the course of the study (*P *< 0.001), from 100% through to Week 4, to 99.1% (108/109) in Weeks 5-8, and to 89.0% (97/109) in Weeks 9-12. When stratified by pre-study DMD, 94.0% (63/67; im IFN β-1a), 100% (14/14; GA), 100% (25/25; sc IFN β-1b) and 100% (3/3; sc IFN β-1a) of patients in the completers' population were adherent to treatment. All scheduled injections were administered by 73.4% (80/109) of patients over the 12-week study period. *Post hoc *analysis of adherence in the ITT population, based on expected number of planned injections prior to withdrawal, revealed that 91.6% (109/119) were adherent to treatment throughout study duration.

Mean (SD) baseline PASAT score was similar in patients who were adherent (42.67 [10.9], n = 102) and those who were not adherent (42.86 [13.0], n = 14) to treatment. No significant correlation between adherence and baseline PASAT score was observed (Spearman's correlation: ρ = -0.011; *P *= 0.909). No significant predictors of treatment adherence or tolerability were identified using logistic regression analysis for each baseline variable (data not shown). Mean HADS depression or anxiety scores did not differ significantly from baseline at Weeks 4, 8 and 12 (*P *= 0.482 and *P *= 0.156, respectively).

Overall, 35.6% of missed injections (31/87) were due to medical reasons (patients experienced AEs that precluded injection), 20.7% (18/87) due to forgetfulness and 27.6% (24/87) due to 'other reasons'. Pain at the injection site and 'problems using the autoinjection device'/'device unusable' accounted for 3.4% (3/87) and 6.9% (6/87) of missed injections, respectively. At Weeks 4 and 8, most injections were missed due to medical reasons (42.3% [11/26] and 40.1% [11/27], respectively), while at Week 12 most were due to 'other' reasons (42.4% [14/33]) and medical reasons accounted for 27.3% [9/33] of missed injections.

### Evaluation of the injection process

Mean MSTCQ scores improved significantly from Week 4 to 12 for the FLS (*P *= 0.022) and GSE (*P *= 0.002) domains, but not for ISR (Table 2). However, a *post hoc *analysis revealed a significant positive correlation between FLS at Week 8 and HADS scores at baseline and Weeks 4, 8 and 12 for anxiety (Pearson's correlation: ρ = 0.203-0.298; *P *< 0.05) and depression (Pearson's correlation: ρ = 0.212-0.330; *P *< 0.05). A positive correlation was also seen between GSE at Weeks 4, 8 and 12 and HADS scores for anxiety and depression at all study visits (*P *≤ 0.001; Pearson's correlation: ρ = 0.301-0.495 at Week 4, 0.340-0.519 at Week 8 and 0.322-0.490 at Week 12).

**Table 2:** Summary of changes in MSTCQ scores from Week 4 to 12 in the intent-to-treat population (n = 119)

Time point	ISR*	FLS*	GSE^†^
Week 4 (n = 115)			
Mean (SD)	13.2 (4.02)	13.4 (4.56)	6.8 (3.16)
Median (range)	13 (4-19)	13 (6-20)	6 (3-15)
Week 8 (n = 109)			
Mean (SD)	13.4 (3.64)	12.9 (4.10)	7.5 (3.16)
Median (range)	13.5 (6-20)	12 (5-20)	8 (3-15)
Week 12 (n = 104)			
Mean (SD)	13.1 (3.39)	12.2 (4.30)	7.6 (3.05)
Median (range)	13 (6-20)	11 (5-20)	8 (3-15)
p-value^‡^	0.902	0.022	0.002

*Range: 1 (mild) to 20 (severe)

^†^Range: 1 (poor treatment satisfaction) to 15 (excellent treatment satisfaction)

^‡^Repeated measures analysis of variance

*FLS *'flu-like' symptoms, *GSE *global side effects, *ISR *injection-site reactions; *MSTCQ *Multiple Sclerosis Treatment Concern Questionnaire, *SD *standard deviation

Pain at injection site (mean [SD] VAS score) significantly increased over time (*P *< 0.001; repeated measures ANOVA) from 15.7 (18.82; n = 116) at Week 4 to 28.4 (27.37; n = 109) at Week 8 and to 31.2 (27.57; n = 107) at Week 12. Pain upon injection (mean [SD] MSTCQ score) also increased significantly (*P *< 0.001) from 2.1 (0.99) at Week 4 to 2.55 (1.05) at Week 8 and to 2.49 (1.03) at Week 12. The mean (SD) number of ibuprofen tablets taken decreased from 23.4 (8.60) at Week 4 to 18.7 (11.03) at Week 8 and 13.9 (11.77) at Week 12. There was no significant correlation, however, between the number of ibuprofen tablets taken and VAS score for pain at injection site at Weeks 4, 8 and 12 (*p *> 0.398, respectively).

### Overall device evaluation

Based on patient experience, overall convenience was consistently reported as the most important benefit of the electronic autoinjection device at Weeks 4 (38.5%; 40/104), 8 (42.3%; 41/97) and 12 (44.6%; 41/92; Additional File 1: Figure S2). The second most important benefit of the autoinjection device was reported to be less injection pain (24.0%; 25/104) at Week 4 and fewer FLS at Weeks 8 (19.6%; 19/97) and 12 (23.9%; 22/92). When stratified according to pre-study DMD, less injection pain was reported as the most important benefit by patients receiving pre-study sc IFN β-1b (40.0%; 10/25) and fewer FLS by patients receiving sc IFN β-1a (66.7%; 2/3) at Week 4. Convenience and fewer FLS were each reported as the most important benefit by 50.0% (1/2 and 1/2, respectively) of the patients receiving sc IFN β-1a at Week 12. Patients receiving pre-study GA reported fewer ISRs as the most important benefit at Week 12 (50.0%; 4/8) and as the second most important benefit at Week 8 (36.4%; 4/11). Patients receiving pre-study im IFN β-1a consistently reported convenience as the most important benefit, and fewer FLS as the second most important benefit, at Weeks 4, 8 and 12.

At Week 12, patients rated the autoinjection device as highly convenient to use, with an overall median evaluation score of 1.4 (range 1.2-1.9). Most patients thought it was 'very easy' to change the multidose cartridge (74.1% [80/108]); to inject using the device (62.6% [67/107]); to store (61.7% [66/107]), transport (57.5% [61/106]) and hold (56.1% [60/107]) the device; and to both attach (55.1% [59/107]) and detach (55.1% [59/107]) the needle. Less than half of patients thought it was 'very easy' to change the comfort settings (47.1% [48/102]) and the batteries (35.1% [33/94]), and that the device was 'very' attractively designed (43.8% [46/105]) and silent (43.5% [47/108]).

### Safety

A total of 432 AEs were reported; 96.8% (418/432) were mild or moderate in severity, 60.6% (262/432) were considered to be treatment related, 1.6% (7/432) resulted in temporary interruption of treatment and 1.6% (7/432) in discontinued use of the study drug. Of the patients who withdrew from the study because of AEs, one did so due to an injection-related AE (injection-site pain), which presented in association with fatigue. Five patients withdrew from the study for non-device-related reasons (dose adjustment [1 patient], pregnancy [2 patients] and DMD-related toxicity [2 patients]). The most frequent AEs were FLS, headache and ISRs (Table 3). One (0.2%) SAE was reported, which required hospitalization of the patient and was considered to be related to the study drug (elevated liver enzymes).

**Table 3:** Total treatment-emergent adverse events (intent-to-treat population, n = 119)

Adverse event	n (%)
'Flu-like' symptoms	170 (39.4)
Headache	84 (19.4)
Injection-site reaction	46 (10.6)
Gastrointestinal abnormalities	25 (5.8)
Pain	20 (4.6)
Anxiety	13 (3.0)
Infections	10 (2.3)
Hypersensitivity reaction	7 (1.6)
Cytopenia	7 (1.6)
Hepatic disorders	7 (1.6)
Vertigo	6 (1.4)
Upper respiratory tract infection	5 (1.1)
Tachycardia	4 (0.9)
Dysmenorrhoea	3 (0.7)
Other	25 (5.8)
Total	432 (100)

## Discussion

The objective of this study was to assess short-term adherence and tolerability of sc IFN β-1a, self-administered using an electronic autoinjection device, in patients with RRMS who had switched from another injectable DMD or a different injection system for sc IFN β-1a. Over 85% of patients were adherent to treatment over the 12-week course of the study, as recorded by the device dosing log. When adherence was adjusted to account for missed injections by patients who withdrew from the study for non-device-related reasons, adherence was 92%. The high adherence rates reported in this study are consistent with findings of a previous study, in which most patients found the same autoinjection device to be suitable or very suitable for self-injection of sc IFN β-1a [14]. Notably, this earlier study did not assess adherence, as recorded objectively by the device dosing log.

Adherence did not correlate with baseline cognitive function (measured using the PASAT) and no predictors of adherence were identified. The absence of identified predictors may reflect certain characteristics of the study population: most patients were under 40 years of age with low levels of disability and very good cognitive function. Overall, the safety profile of IFN β-1a serum-free formulation in the current study was consistent with previous observations with the original IFN β-1a formulation [23-25].

Adherence rates were very high at the start of the study but decreased as the study progressed. This may have been due to patients experiencing IFN-specific AEs once the drug was administered at full dose, particularly FLS, one of the most common AEs associated with IFN therapy. However, the most common reason for missing injections changed from medical reasons at Weeks 4 and 8 to 'other' reasons at Week 12. Forgetfulness, one of the most common reasons for missed injections [5], was responsible for 21% of missed injections overall and may be particularly problematic in patients with impaired cognitive function due to MS. Baseline cognitive function was very good in the majority of patients and did not predict adherence in this study. However, the PASAT test, which was used to assess cognitive function in this study, measures attention and working memory rather than the long-term memory skills that are required for processes such as remembering to perform regular injections, so the lack of association between PASAT score and adherence may not be surprising. Therefore, no conclusions could be drawn regarding the relation between cognitive function and adherence using the autoinjection device. In addition, to what extent different cognitive functions influence adherence in general is not fully understood. Furthermore, it should be considered that patients may prefer to give 'forgetfulness' as the reason for missing one or more injections rather than other reasons which may be considered more problematic by their neurologist, such as treatment fatigue.

No significant baseline predictors of adherence were identified; however, depression may have influenced treatment outcome. Depression, a common symptom of MS, may reduce motivation, which may in turn reduce adherence. Indeed, depression has previously been reported to be a cause of treatment discontinuation [8]. No significant correlation between depression and adherence was observed in this study, however, and a longer study period would be required to explore the relation between depression and adherence to MS therapy. The positive correlation between baseline HADS scores and reported FLS and GSE over the course of the study suggests that depression and anxiety may increase the extent to which patients are aware of side effects and also their perception of the magnitude of such events.

The high adherence rates in this 12-week study are encouraging, particularly as the use of an autoinjection device to collect adherence data likely provides a more reliable indication of adherence to treatment than other measures of adherence such as MPR or retrospective self-reporting by patients used in other studies [3,26,27]. The high level of adherence may indicate that most patients found the device convenient and easy to use. Convenience, less injection pain and fewer FLS were rated by patients as the most important benefits of the autoinjection device. Overall, convenience was reported as the main benefit throughout the study, consistent with previous studies of this device [14,28]. Notably, device features associated with handling and ease of use were rated highly. The majority of patients found it 'very easy' to change the multidose cartridge and needle, hold the device and perform the injection. Simplification of the injection process, for example through the use of an autoinjection device that only needs to be loaded once a week, may encourage patients to perform injections and alleviate treatment fatigue. Such features may also be particularly beneficial to patients who have impaired dexterity due to MS [2], increasing treatment satisfaction and independence. The majority of patients also thought the device was 'very easy' to transport, which may assist patients with MS to adhere to treatment while travelling and on holiday.

Patients considered 'less injection pain' to be the second most important benefit of the autoinjection device at Week 4 and the third most important benefit at Weeks 8 and 12. As the study progressed, patients reported an increase in both pain at the injection site and pain upon injection. Repeated injections may cause progressive damage (e.g. nodules, bruises) to sc tissue, thus increasing injection pain. These side effects may be particularly problematic to patients who are unaccustomed to sc injections, such as patients who switched from im IFN β-1a, which comprised 63% of our study population. As data regarding injection-related pain with prior treatment were not collected, it is not known how injection pain during the study compared with previous injection experience.

All injectable DMDs are associated with ISRs; however, symptoms and reaction severity vary depending on the drug and the route of administration. ISRs are more commonly associated with sc rather than im injections [8]. As 63% of patients in this study switched from im to sc injections, an increase in ISRs in either this subset of patients or the total patient population would not be unexpected. Mean ISR score, however, did not change from baseline over the course of the study, suggesting that patients did not note an increase in ISRs when switching to sc injection. Despite this, 'fewer ISRs' was reported as the most important benefit of the device by 7% of patients in this subgroup at Week 8. 'Fewer ISRs' was rated as the most important benefit of the device at Week 12, and second most important benefit at Week 8, by patients previously receiving sc GA therapy. ISRs are the most common side effect associated with GA injections [29], therefore the reported reduction may have been due to a switch in both DMD and device.

Overall, patients reported a decrease in FLS over the course of the study despite a concurrent reduction in ibuprofen usage, and 'fewer FLS' was reported as the second most important benefit at Weeks 8 and 12. However, it should be noted that the autoinjection device is unlikely to be implicated in the observed reduction in FLS. As discussed previously, pre-study DMD therapy would have likely influenced AEs experienced during the study. FLS are one of the most common AEs associated with IFN β therapy, which was being received by 87% of patients prior to the study. No change in FLS during the study would have been expected in patients receiving pre-study sc IFN β. FLS may have worsened in some patients previously receiving im IFN β-1a owing to increased IFN dose and injection frequency [30]; however, increased injection frequency may favour tachyphylaxis, therefore reducing the presence and severity of FLS (authors' personal observations) [31]. Indeed, fewer FLS were consistently reported as the second most important benefit of the autoinjection device by patients receiving pre-study im IFN β-1a at Weeks 4, 8 and 12. Patients switching from GA would have been unaccustomed to FLS, which are particularly prevalent in the first few weeks of treatment with IFN β [8,32] and occur most frequently for the first 6 months. In addition to FLS, global side effects improved over the course of the trial. As ibuprofen usage was mandatory for the first 4 weeks of the study, whether reduction in ibuprofen usage was a result of reduced AEs is unknown. Recent reports on side effects related to ibuprofen use, however, may suggest that its usage should be adjusted according to the presence and severity of side effects [33].

Limitations of the study should be considered when interpreting the study findings. At 12 weeks, the study was only able to assess short-term adherence; however, we are exploring the possibility of assessing safety, tolerability and treatment adherence/persistence after longer-term follow-up in this patient population. Absence of data on adherence to previous medication and pre-study or baseline injection pain precludes inferences on the benefits of switching to the autoinjection device with respect to adherence and injection pain. In addition, patient perception and reporting of pain is subjective. Pre-study DMDs were not equally represented among patients, with the majority switching from im IFN β-1a; this imbalance limited the conclusions that could be drawn regarding the influence of previous treatment on subsequent outcomes. For example, pre-study DMD may have influenced AEs, patient perception of injection-related AEs and, therefore, opinion of the autoinjection device. Finally, there was no control group in this observational study, so we were not able to discriminate between the effects of the new device and those of changing treatment in patients who had switched to sc IFN β-1a from a different DMD at study entry. However, a previous international study of this new device recruited only patients already being treated with sc IFN β-1a [14]; therefore, the effects of changing device have only previously been reported for some of the parameters investigated in the current study.

## Conclusions

In conclusion, the convenience and ease of use of an electronic autoinjection device may increase adherence in patients with RRMS, allowing patients to receive the full benefits of DMD therapy.

## Abbreviations

AE: Adverse event; ANOVA: Analysis of variance; BRIDGE: RebiSmart to self-inject Rebif serum-free formulation in a multidose cartridge; CI: Confidence interval; DMD: Disease-modifying drug; EDSS: Expanded Disability Status Scale; ET: Early termination; FLS: 'flu-like' symptom; GA: Glatiramer acetate; GSE: Global side effect; HADS: Hospital Anxiety and Depression Scale; IFN: Interferon; im: Intramuscular; ISR: Injection-site reaction; ITT: Intent-to-treat; MPR: Medication possession ratio; MS: Multiple sclerosis; MSTCQ: MS Treatment Concern Questionnaire; PASAT: Paced Auditory Serial Addition Task; RRMS: Relapsing-remitting multiple sclerosis; SAE: Serious adverse event; sc: Subcutaneous(ly); SD: Standard deviation; TEAE: Treatment-emergent adverse event; tiw: Three times weekly; VAS: Visual analogue scale.

## Competing interests

AL has been an advisory board member for Biogen Idec, Merck Serono and Bayer Schering; has received travel grants and honoraria from Bayer Schering, Biogen Dompé, Merck Serono, Novartis, Sanofi-Aventis and Teva; has received research grants from Bayer Schering, Biogen Dompé, Merck Serono, Novartis and Sanofi-Aventis; has received travel and research grants from the Associazione Italiana Sclerosi Multipla; and is a consultant for "Fondazione Cesare Serono". CF has received personal compensation for activities and research from Biogen Dompé, Bayer Schering, Merck Serono and Sanofi-Aventis. VB-M has received travel grants from Bayer Schering, Biogen Dompé, Merck Serono, Novartis and Sanofi-Aventis. SC has received personal compensation from CIC International for serving as a member of an editorial advisory board; and financial support for research activities from Biogen Dompé. PB has no competing interests to declare. MC has received personal compensation for activities as a speaker from Bayer AG. DC has received personal compensation for activities with Teva Neuroscience, Novartis, Sanofi-Aventis and Merck Serono; research support from Sanofi-Aventis, Bayer Schering Pharma, Merck Serono, Novartis and Teva Neuroscience; compensation and/or research work has been funded, entirely or in part, by a grant to his university. The grant agreement requires that the name of the funding entity and the purpose of the grant may not be disclosed. The funding entity is a governmental organization. AU has received financial support for research and honoraria for consultation, speaking at meetings, or both, from Genentech, Roche, Allergan, Merck Serono, Sanofi-Aventis, Biogen Idec, Biogen Dompé, Bayer Schering, Novartis, and the Consortium of Multiple Sclerosis Centers. MdI has no competing interests to declare. GDL has received travel grants from Biogen Dompé and Merck Serono; and speaker fees from Novartis and Sanofi-Aventis. AM has received consulting fees from Merck Serono. AP is a salaried employee of Merck Serono S.p.A., Rome, Italy.

## Authors' contributions

AL: participated in the design of the study and in interpretation of the results, helped to draft and revise the manuscript. CF: participated in study coordination (patient recruitment), approved the manuscript at each stage of development. VB-M: participated in study coordination (patient recruitment), approved the manuscript at each stage of development. SC: participated in study coordination (patient recruitment), approved the manuscript at each stage of development. PB: participated in study coordination (patient recruitment), approved the manuscript at each stage of development. MC: participated in study coordination (patient recruitment), approved the manuscript at each stage of development. DC: participated in study coordination (patient recruitment), approved the manuscript at each stage of development. AU: participated in study coordination (patient recruitment), approved the manuscript at each stage of development. MdI: participated in study coordination (patient recruitment) and the collection of clinical data, approved the manuscript at each stage of development. GDL: participated in study coordination (patient recruitment) and the collection of clinical data, approved the manuscript at each stage of development. AM: performed the statistical analysis and helped to draft the manuscript. AP: participated in the design of the study and in interpretation of the data. All authors read and approved the final manuscript.

## Previous presentation

Presented as poster (poster number 427) at XLI Congress of the Italian Society of Neurology (Società Italiana di Neurologia); 23-27 October 2010

## Pre-publication history

The pre-publication history for this paper can be accessed here:

http://www.biomedcentral.com/1471-2377/12/7/prepub

## Supplementary Material

Additional file 1 **Figure S1**. Type and scheduling of assessments during the 12-week study. **Table S1**. Baseline characteristics (intent-to-treat population). **Figure S2**. Most important benefit of the electronic autoinjection system as rated by patients at (a) Week 4, (b) Week 8 and (c) Week 12 (intent-to-treat population). IFN, interferon; im, intramuscular; sc, subcutaneous. Click here for file
