# Supplementary material for: Patient adherence to and tolerability of self-administered interferon β-1a using an electronic autoinjection device: a multicentre, open-label, phase IV study
Source: BMC Neurol. 2012 Mar 5;12:7. doi: 10.1186/1471-2377-12-7 (PMC3368780; doi:10.1186/1471-2377-12-7)
Supplement: Additional file 1 — Figure S1. Type and scheduling of assessments during the 12-week study. Table S1. Baseline characteristics (intent-to-treat population). Figure S2. Most important benefit of the electronic autoinjection system as rated by patients at (a) Week 4, (b) Week 8 and (c) Week 12 (intent-to-treat population). IFN, interferon; im, intramuscular; sc, subcutaneous. [file 1471-2377-12-7-S1.DOC]

**Supplementary Table 1.** Baseline characteristics (intent-to-treat population).

| Characteristic |  | N=119 |
| --- | --- | --- |
| Age, years | Mean (SD) | 37.9 (9.68) |
|  | 95% CI | 36.2–39.7 |
|  | Median (range) | 39 (18–58) |
| Sex, n (%) | Female | 90 (75.6) |
|  | Male | 29 (24.4) |
| Duration of MS, years | Mean (SD) | 5.8 (5.31) |
|  | 95% CI | 4.9–6.8 |
|  | Median (range) | 5 (0–22) |
| EDSS score^*^ | Mean (SD) | 2.1 (1.2) |
|  | 95% CI | 1.9–2.3 |
|  | Median (range) | 2 (0–6) |
| PASAT score^†^ | Mean (SD) | 42.7 (11.1) |
|  | 95% CI | 40.6–44.7 |
|  | Median (range) | 45 (5–60) |
| HADS anxiety score^‡^ | Mean (SD) | 7.4 (4.6) |
|  | 95% CI | 6.6–8.3 |
|  | Median (range) | 7 (0–20) |
| HADS depression score^‡^ | Mean (SD) | 5.6 (4.3) |
|  | 95% CI | 4.8–6.4 |
|  | Median (range) | 5 (0–20) |

CI, confidence interval; EDSS, Expanded Disability Status Scale; HADS, Hospital Anxiety and Depression Scale; MS, multiple sclerosis; PASAT, Paced Auditory Serial Addition Task; SD, standard deviation.

^*^Range: 0 (no disability) to 10 (severe disability resulting in death).

^†^n=116; range: 0 to 60 (higher scores indicate better cognitive function).

^‡^Range: 0 to 21 (higher scores indicate more severe symptoms).

**Supplementary Figure 1.** Type and scheduling of assessments during the 12-week study.

AEs, adverse events; e-CRF, case report form; HADS, Hospital Anxiety And Depression Scale; MSTCQ, Multiple Sclerosis Treatment Concern Questionnaire; PASAT, Paced Auditory Serial Addition Task; sc, subcutaneous; SD, study day; tiw, three times weekly; V, visit; W, week.

**Supplementary Figure 2.** Most important benefit of the electronic autoinjection system as rated by patients at (a) Week 4, (b) Week 8 and (c) Week 12 (intent-to-treat population). IFN, interferon; im, intramuscular; sc, subcutaneous.
